# Factors influencing access of HIV and sexual and reproductive health services among adolescent key populations in Kenya

**DOI:** 10.1007/s00038-020-01373-8

**Published:** 2020-04-26

**Authors:** Kimathi Robert, Mireku Maryline, Kyongo Jordan, Digolo Lina, Musyoki Helgar, Ikahu Annrita, Mukoma Wanjiru, Otiso Lilian

**Affiliations:** 1 LVCT Health, Nairobi, Kenya; 2 National AIDS and STIs Control Program (NASCOP), Nairobi, Kenya

**Keywords:** Adolescents, Adolescent key populations, People who inject drugs, Sex workers, Men who have sex with men (MSM)

## Abstract

**Objectives:**

The objective of this study is to identify enablers and barriers in access of HIV and sexual reproductive health (SRH) services among adolescent key populations (KP) in Kenya.

**Methods:**

A cross-sectional study using qualitative methods was conducted between October 2015 and April 2016. A total of 9 focus group discussions and 18 in-depth interviews were conducted with 108 adolescent KPs in Mombasa, Kisumu and Nairobi Counties of Kenya. Data were recorded digitally, translated, transcribed and coded in NVivo10 prior to analysis.

**Results:**

Adolescent KPs preferred to access services in private health due to increased confidentiality, limited stigma and discrimination, access to adequate amount of condoms, friendly and fast-tracked services. Negative health provider attitudes made adolescent KPs dislike accessing health care in public health facilities. There was a lack of adolescent key population’s policies and guidelines on HIV and SRH.

**Conclusions:**

The study has demonstrated existing enablers and barriers to provision of HIV/SRH services for an at-risk population for which limited data exist. The results provide a basis for program redesign involving the adolescent KPs to minimize barriers for access to HIV/SRH services.

## Introduction

According to the 2018 Joint United Nations Programme on human immunodeficiency virus (HIV)/acquired immunodeficiency syndrome (AIDS)—(UNAIDS) global update, there were 1.8 million new HIV infections worldwide in 2017 (UNAIDS 2018). This number of new infections is indicative of significant progress from 1996 when the number of new infections was 3.4 million but is still way off the target of half a million new infections in the year 2020 (UNAIDS 2018). The majority (66%) of these new infections were in sub-Saharan Africa, specifically Eastern and Southern Africa (45%) of global new infections (UNAIDS 2018). The total number of people living with HIV in 2017 was 36.9 million with 53% of them living in Eastern and Southern Africa (UNAIDS 2018). Gay men and other men who have sex with men accounted for an estimated 57% of new HIV infections in Western and Central Europe and North America in 2017, an estimated 41% of new infections in Latin America, more than 25% of new HIV infections in Asia and the Pacific and the Caribbean, about 20% of new infections in Eastern Europe and Central Asia and the Middle East and North Africa and an estimated 12% of new infections in Western and Central Africa (UNAIDS 2018). In Kenya, HIV prevalence among key populations was estimated to be 18.2% for men who have sex with men (MSM), 29.3% for female sex worker (FSW) and 18.3% for people who inject drugs (PWID), while that of the general population was 5.6% in 2015 (NACC 2015).

Adolescents still face numerous challenges in the area of sexual and reproductive health, which hinder their utilization of available Sexual and Reproductive HealthCare Services (Aninanya et al. 2015). Adolescents face further obstacles in accessing and utilizing sexual reproductive health services owing to the influence of social constructions of masculinity and sexual orientation, which has a bearing on how they view sexual and reproductive health services and their use (Shabani et al. 2018). Key populations have poor access to services in regions where there are prohibitory laws and policies (World Health Organization 2014; Bekker and Hosek 2015). Same sex intercourse, especially for adolescent, presents major barriers toward implementing effective HIV and sexual reproductive health interventions, policies, due to other health problems attributable to high-risk behavior or their developmental stage, or a combination of both (Delany-Moretlwe et al. 2015).

Available evidence shows that MSM who disclose their orientation, through choice or necessity, report family rejection, public humiliation, harassment by authorities and ridicule by health-care workers (Ayehu et al. 2016; Muthengi et al. 2016). This population often face significant challenges in accessing HIV care and treatment (Mark et al. 2017) due to stigma and discrimination from health providers (Godia et al. 2013), and ignorance within health systems (Johnston et al. 2017) about their sexual orientation (World Health Organization 2014). Inadequate health-care providers’ skills have been identified as a key barrier to provision of services to key populations (Baggaley et al. 2015). In some regions, access to services for key populations has improved with specialized drop-in centers and an increase in service provision for key population in public health centers on the rise (Dhana et al. 2014). Unfortunately, these services, in many cases like in Kenya, do not address the needs of the adolescent key populations (Goldblatt et al. 2015). Sex work is illegal in Kenya, and adolescent sex workers regularly experience violence from the public and police, rape and abuse by clients (Ssewanyana et al. 2018). In addition, sex workers are generally stigmatized, marginalized and their activities considered as illegal by the societies in which they live increasing their vulnerability (Ziraba et al. 2018) to HIV and impeding efforts to reach them with sexual reproductive health, HIV/AIDS information and services (Kurth et al. 2015).

There exist inadequate policy and legal frameworks (Delany-Moretlwe et al. 2015) hindering planning, programming and development of programs (Mutumba and Harper 2015) that impact specifically on the health of adolescent key populations (Delany-Moretlwe et al. 2015). Data are not adequately disaggregated by age, gender and risk behavior to aid in planning at national and sub-national levels (Johnston et al. 2017). In addition, policy and programming for adolescent key populations are hampered by the fact that the behaviors they engage in such as sex work (Wanje et al. 2017) and same sex relations are considered illegal in Kenya (Ssewanyana et al. 2018). The aim of this study was to assess the programmatic and policy environment and determine the barriers and opportunities for providing HIV/SRH services to adolescent key populations in Kenya.

## Methods

### Study design and sites

This study employed an exploratory cross-sectional design using quantitative and qualitative methods. This paper addresses only the qualitative aspects of the study to explore the perception f the adolescents on their access to services. The study was conducted between October 2015 and April 2016 in Nairobi, Mombasa and Kisumu counties in Kenya.

These counties were purposively selected as they have the highest HIV burden among young people and key populations in Kenya. These counties were also sampled to provide geographic, cultural, epidemiologic and HIV programming variations.

### Study population

Study participants included boys: aged 10–19 years and self-reporting engagement in same sex relations; girls aged 10–19 years and engaging in sex work; and male and female adolescents who inject drugs. We recognize that adolescents less than 18 years are not officially recognized as sex workers but are children exploited for sex. However, for the sake of standardization and ease of reference we classified them as sex workers. Only those who gave consent were invited to participate in the study. Other study participants included health policy makers at national and county levels, key populations program implementers and health service providers who gave consent to participate in the study.

### Sampling

The adolescents participating in the in-depth interviews (IDIs) and focus group discussions (FGDs) were purposively sampled and selected based on their self-reporting as either engaging in adolescent boys who have sex with boys and men, injection drug. Primarily self-supporting, lived independently and often had no parental control.

Health programmers were selected from organizations which implemented or advocated for youth friendly services in the study sites. Decision makers were sampled from National AIDS and STIs Control Program (NASCOP), representatives from national technical working groups and key population organizations, county governments, civil society organizations, Ministry of Education and development partners who fund youth and HIV programmes.

Sampling for qualitative data collection was guided by evidence on the requirements to reach saturation, especially by considering the homogeneity of the populations and the proposed numbers of FGDs and IDIs. Content analysis was carried out concurrently with the data collection to track data saturation.

### Data collection

Qualitative data were collected through semi-structured interviews with adolescent key populations, health providers, programmers and decision makers. Data collection was conducted by trained interviewers who are not affiliated with the clinical services provided at the sites or programmes involved in the study to reduce social desirability bias. The data collection tools for the interviews and FGDs were translated into Swahili and piloted prior to data collection to test validity of the tools. Interviewers with experience and training in research ethics and methods administered all the tools. The in-depth interviews and focus group discussions were audio recorded. To ensure participant confidentiality, data collected for this study were coded using study identification numbers in place of participant’s names.

In-depth interviews and focus group discussions were conducted with adolescents’ boys (10–19 years) engaging in same sex relations; girls aged 10–19 years and engaging in sex work; and (10–19 years) male and female adolescents who inject drugs to better understand their experiences with available HIV/sexual and reproductive health (SRH) health services. The interviews and FGDs explored issues of availability, access, appropriateness and friendliness of health services. Focus group discussions explored group perceptions while individual in-depth interviews focused on personal experiences.

Interviews with health providers focused on their experiences with providing services to adolescent key populations, while exploring access of the HIV/SRH services available, perceptions of their clients’ experiences and recommendations for improvement in HIV/SRH services.

Policy makers and development partners’ interviews explored policy environment for delivery of services to adolescents, opportunities and barriers for delivery of services among adolescents, including opinions on delivery and access of HIV/SRH services to adolescents and the technical considerations required in service delivery.

### Data analysis

Recorded information was transcribed verbatim and translated into English for the final transcripts. Grounded theory approach guided the analysis of data. Themes were developed deductively from the literature and inductively from reading information in the transcripts. A coding framework was then developed from the generated themes, and transcripts were imported into NVivo 10 QSR software for management and analysis.

## Results

Results are presented by the three key thematic areas from the analysis:

1. Availability and utilization of HIV and SRH services;
2. Barriers to uptake of services by adolescent KPs;
3. Adolescent KPs’ HIV and SRH policy and legal environment.

### Availability and utilization of HIV and SRH services

Most health providers in the study reported providing health services to adolescent KPs that accessed the health facility. These services included: HIV testing, counseling and treatment; sexually transmitted infections screening and treatment; health education; family planning; voluntary medical male circumcision; condoms and lubricants provision; and referrals to specialized facilities where applicable.

Many adolescents engaging in sex work mentioned confidentiality from service providers as a core element in the access to services. The nature of their work made them frequent facilities due to the need for services such as sexually transmitted infections (STI) treatment, and this made them prefer facilities that could offer STI treatment, provide contraceptives, uphold confidentiality, treat them with dignity and were affordable.

Their service was not bad; they explain to you very well and give you freedom to choose in case you are undecided, their services were good. [Adolescent Sex Worker-Mombasa]

It is not good, when you go there you will be discriminated because you are dirty and look like a thief, and again you are kept there for long as they serve other patients…but they do this because they have not been sensitized about drug users or they don’t have experiences with drug users. [Adolescent drug user -Nairobi]

Policy makers identified health needs of adolescent key populations as health education, guidance and counseling, social support, access to friendly HIV and sexual reproductive health services (condoms, HIV testing, counseling and treatment) drug education particularly treatment and rehabilitation. Similar to policy makers, the health providers identified health needs of adolescent key populations as sexually transmitted infections (STI) and tuberculosis (TB) screening and treatment, HIV testing and counseling, family planning, condom and lubricants distribution, health information, provision of clean needles and syringes, detoxification and rehabilitation, especially for those using drugs. A policy maker described the services required by adolescent who use drugs from psychosocial support to treatment and rehabilitation:

Adolescents who report drug use they need …. health education, they need to be educated on the effects of taking these drugs…. they also need some basic counselling on socio support to help them address their own challenges academic, family related challenges or those who are already using them they need pyscho-social to help them stop using and also those who are already addicted they need to go through rehabilitation and treatment so that they can now be in recovery… [Policy Maker-Kisumu].

Adolescents who use drugs described how lack of money to buy needles put them at risk of HIV. Some reported that they went for services in organizations that provided them with needles which is a priority services they required:

…they bring us needles even in our base and there is no sharing because in the past you could get money for the drugs but lack the needle and it would force you to share… and you don’t know the status of your friend whether they are infected and you will be infected just because of lack 20 bob [shillings] for buying a needle… [Female Injecting Drug User-Mombasa].

### Barriers to uptake of services by adolescent KPs

In spite of availability of services in government facilities, some adolescent key populations felt the services did not meet their needs, were discriminatory or lacked confidentiality. Negative health provider attitudes displayed in public health facilities were reported to be an obstacle in access as noted in the following comments from a focus group discussion:

Public hospitals discriminate and stigmatize us when we go seeking for health services. Health providers’ attitude is very poor towards MSM [Adolescent MSM – Nairobi]

In some hospitals, the health providers will treat MSM like any other patient but once I leave the room, he or she will go and start discussing about my sexuality with his or her colleague. Other health care providers condemn us right in our faces unlike private hospitals where we get confidentiality. [Adolescent female sex worker – Nairobi]

Initially they never reacted negatively then after that they told me to come back again after some time, when I came back I was told to wait for long that I will be attended to later. [Adolescent MSM – Kisumu]

Some health providers acknowledged that adolescent KPs uptake of services was not good and suggested that stigma may be a factor that affects uptake of services:

okay accessibility of services to the adolescents in the clinic is not hundred percent and it is limited and if sometimes we go out there to know why they don’t want to really come out for the services. Some feel shy some feel stigmatized you know they have a lot of unknown fear. [Health service provider - Mombasa]

Some of the health providers/organizations where adolescents accessed services reported that the health services they offered were not always adequate resulting in the need to refer as stated by a health service provider from Nairobi:

Currently we do not offer HIV treatment and again since we do not have laboratory services, we only treat symptomatically and therefore if we find a complex case that needs lab test, we always refer [Health service provider - Nairobi]

Policy gaps that affected access and utilization of services were described by some health workers, specifically the lack of legal provision to offer SRH services to under 18 years which may affect the health facilities’ ability to offer the required services.

we still have a gap in the provision of adolescent services…….where probably they are termed as still children who should be under the care of a parent or a guardian but in real sense these are mature minors because they already know. If you see someone who is already selling sex, is able to talk and negotiate for sex, this is somebody you can reason with and give services regardless of the guardian and parent being absent [Health service provider - Nairobi]

### Health-care provider capacity as a barrier to service uptake

Emphasis was given by respondents in the study on the importance of health-care providers’ capacity in dealing with HIV/SRH needs of adolescent KPs to create a friendly environment for adolescent KPs. Health-care providers mentioned that many of them lacked adequate skills and knowledge about health needs of adolescents. High staff turnover was also reported to be an impediment in the event that those trained left the facility resulting in a skill gap.

Minimal capacity to deal with adolescents was reported by health providers from both public health facilities and NGO facilities. Some of the providers reported that they had not benefited from the trainings as they were perceived to be offered to one cadre (HIV testing counselors):

…and you find that in this adolescent MSM you find that it is only the VCT (Voluntary Counseling and Testing) counselors that normally go for these trainings. I think it should be, you know, open to all the cadres too like the clinical officers, the nurses, the clerks because they are the ones that attend to them first when the client comes to the clinic. So they should also be trained on how to handle them, it should not be specific for counselors… unless you are properly trained and you have proper information, it is a barrier because you won’t attend to this client positively and efficiently [Health Care Provider-Mombasa].

Adolescent KPs reported stigma and discrimination and lack of skills from health service providers who served them and felt there was a need for the providers to be sensitized on how interact with them. This was more common in public health facilities which made them not seek services from them in as much as they were located nearer to them:

For me they discriminate because for my case I was made to wait from 8:00 am to 1:00 pm and I was the first one in the line, she kept on saying she is busy attending to other patients and when I went to her and asked she told me to go and come back again on Tuesday. [Adolescent MSM FGD participant – Kisumu]

There are so many organizations for MSMs but they are so far from us, the nearer ones are government hospitals and people don’t want to go there because of discrimination. When health providers know that you have anal STIs they treat you badly until you cannot advice someone else to go there. So both of you will suffer because when they hear about your story they wouldn’t like to undergo the same treatment. So the government hospital providers should be sensitized because some have discrimination and make us not to go to hospitals or to go far yet there are facilities near us [Adolescent FGD participant -Mombasa].

It’s good but at times it’s a bit challenging especially during methadone, those who give it are not well informed about the drug users [Adolescent drug user’ FGD participant -Nairobi].

### Adolescent KPs’ HIV and SRH policy and legal environment

Program implementers and policy makers had varied opinions on the existing policies and guidelines addressing service delivery of HIV and SRH to adolescents in Kenya. Some of the policy makers from the study cited that service delivery was complicated by the lack of a protective or conducive policy-guided environment, inadequate sex education, lack of guidelines on package of care, legal barriers and stigmatization of same sex intercourse and sex work.

There was an expressed need for programs targeting adolescents to be guided to ensure they were capable of meeting adolescents’ needs. Policy makers and health providers all recognized the need to incorporate adolescents’ voices in the program design to ensure they are considered

…in terms of HIV policies, those who are being engaged mostly are the adults, the adolescent are set aside, not given the voice for them to speak. If they are given chance to speak they will give proper programming or NASCOP to help the adolescent [Health care provider- Mombasa].

Respondents generally felt that service provision to adolescent KPs was largely hindered due to the limitations in the existing HIV/SRH policies which did not address services for adolescents aged 18 and below.

…. The government does not recognize or acknowledge the services that adolescents need because we offer services mostly to female sex workers who are 18 years and above due to the legal age requirement in Kenya [Policy Maker-Nairobi].

Lack of legal protection for adolescents engaged in sex work exposed them further to exploitation by government authorities:

Sometimes I will be arrested on Friday and stay in jail for the whole weekend because the court is open on weekdays only. The police in charge will give me the option of either having sex with him so that he can let me go or wait for the court to be back in session. It is a very hurting thing because someone is asking for sex in exchange for my freedom. [Adolescent sex worker -Nairobi].

Respondents felt there was a need for adequate integration of HIV/SRH information into the education system which largely dealt with adolescents. The lack of this integration was seen as a missed opportunity which could have given some of the adolescent KPs information on how to protect themselves from HIV risk.

Of course the most important one is the education system and education is a tool of prevention, but it can only be a tool of prevention if they are learning the right skills and information within that environment. But what you see now, you find that if they are girls who probably sex workers, keeping them in school would probably stop them from engaging in unprotected sex even if they are being paid for sex; using condoms…, [Policy Maker-Nairobi].

## Discussion

The study demonstrates key barriers among adolescents’ key populations to access age-appropriate information, access to condoms, HIV testing and counseling, and essential sexual and reproductive health and treatment services in Kenya. Many of our findings highlight the generalizability of risk factors that have been documented in studies in Kenya and beyond (Nkansah-Amankra and Walker 2012; Baggaley et al. 2015; Ssewanyana et al. 2018). A technical brief (Interagency Working Group on Key Populations 2014) demonstrated that young key populations are more vulnerable to HIV due to widespread discrimination, stigma and violence (Vitek et al. 2014), and the particular vulnerabilities of youth (Godia et al. 2014), power imbalances in relationships and, sometimes, alienation from family and friends (Mutumba and Harper 2015). The high level of stigma prevents their access and utilization of HIV prevention and treatment services (Musyoki et al. 2018). In addition, these young people within key populations often have lower knowledge of HIV risks, or lower ability to mitigate those risks such as negotiating condom use (Dellar et al. 2015), compared with their older, more experienced counterparts (Wanje et al. 2017)

Access to services is influenced by negative health providers attitudes (Godia et al. 2014) with HIV service providers often poorly equipped to serve young key populations, while the staff of programs for young people may lack the appropriate sensitivity, skills and knowledge (Delany-Moretlwe et al. 2015; Shabani et al. 2018)

Policy makers and health providers in our study cited the need to address legal provisions that limit the services that those under 18 years can get in the facilities as the law restricts what health providers can provide including HIV testing and counseling services without parental consent. This resonates with other studies across the globe that calls for targeted investments (Mmari and Astone 2014) and policy reforms in health-care systems (Igras et al. 2014; Ssewanyana et al. 2018). Criminalization of sex work and same sex behavior also restricts access to services that the young KPs require. In this way, young people from key populations are made more vulnerable by policies and laws that demean, criminalize or penalize them or their behaviors, and by education and health systems that ignore or reject them and that fail to provide the information and services, including treatment they need to keep themselves safe (World Health Organization 2014).

Adolescents’ key populations need more routine access to sexual reproductive health services that effectively addresses the developmental, social political, legal and other issues in their holistic life (Igras et al. 2014; Kurth et al. 2015). If these issues are not addressed, the utilization of health services by adolescent key populations will remain low necessitating risky sexual behaviors (Feleke et al. 2013; Ayehu et al. 2016).

### Limitations

The study is limited by a number of different factors; firstly, the study was a rapid assessment with limited scope aimed at supporting policy, programmes and future research for adolescent key populations. Secondly, qualitative study conducted was limited to three city counties in Kenya. Although not representative of the variation in social, epidemiological and geographical contexts of Kenya, results from these three counties could only be generalized to urban areas in Kenya. Lastly the sample size of the population of interest reported was too small to be generalizable (Table 1).

**Table 1:** Adolescent key population demographics; Kenya (2015–2016)

Demographics	Adolescent boys who have sex with boys and men	Adolescent (girls) sex workers	Adolescents who inject drugs—female	Adolescents who inject drugs—male
*Age*				
10–14 years	0	2	0	0
15–19 years	36	35	14	21
Mean age (years)	17	17	18	18
*Education level*				
Primary	6	20	10	12
Secondary	21	15	4	2
Tertiary	9	2	0	0
*Number of children*				
0	35	15	8	16
1	1	20	5	5
2	0	2	1	0
*Marital status*				
Single	35	33	12	18
Married	1	1	2	0
Separated	0	3	0	0

Number of children refers to the biological children under the care of the adolescent key population at the time of study

### Conclusion

Adolescent key populations have pressing needs that require targeted programming that addresses their overlapping vulnerabilities of adolescence and risky behaviors. The presence of diverse private, public and civil society service providers in the country presents existing platforms for improvement in access and capacity of health-care providers. Social and legal environments need to be continuously addressed at different ecological levels of adolescents’ key populations.

## References

[CR1] Aninanya GA, Debpuur CY, Awine T (2015). Effects of an adolescent sexual and reproductive health intervention on health service usage by young people in northern Ghana: a community-randomised trial. PLoS ONE.

[CR2] Ayehu A, Kassaw T, Hailu G (2016). Level of young people sexual and reproductive health service utilization and its associated factors among young people in Awabel District, Northwest Ethiopia. PLoS ONE.

[CR3] Baggaley R, Armstrong A, Dodd Z (2015). Young key populations and HIV: a special emphasis and consideration in the new WHO consolidated guidelines on HIV prevention, diagnosis, treatment and care for key populations. J Int AIDS Soc.

[CR4] Bekker L-G, Hosek S (2015). HIV and adolescents: focus on young key populations. J Int AIDS Soc.

[CR5] Delany-Moretlwe S, Cowan FM, Busza J (2015). Providing comprehensive health services for young key populations: needs, barriers and gaps. J Int AIDS Soc.

[CR6] Dellar RC, Dlamini S, Karim QA (2015). Adolescent girls and young women: key populations for HIV epidemic control. J Int AIDS Soc.

[CR7] Dhana A, Luchters S, Moore L (2014). Systematic review of facility-based sexual and reproductive health services for female sex workers in Africa. Glob Health.

[CR8] Feleke SA, Koye DN, Demssie AF, Mengesha ZB (2013). Reproductive health service utilization and associated factors among adolescents (15–19 years old) in Gondar town, Northwest Ethiopia. BMC Health Serv Res.

[CR9] Godia PM, Olenja JM, Lavussa JA (2013). Sexual reproductive health service provision to young people in Kenya; health service providers’ experiences. BMC Health Serv Res.

[CR10] Godia PM, Olenja JM, Hofman JJ, Van Den Broek N (2014). Young people’s perception of sexual and reproductive health services in Kenya. BMC Health Serv Res.

[CR11] Goldblatt A, Kwena Z, Lahiff M (2015). Prevalence and correlates of HIV infection among street boys in Kisumu, Kenya. PLoS ONE.

[CR12] Igras SM, Macieira M, Murphy E, Lundgren R (2014). Investing in very young adolescents’ sexual and reproductive health. Glob Public Health.

[CR13] Interagency Working Group on Key Populations (2014) HIV and young people who sell sex: a technical brief, pp 1–44

[CR14] Johnston LG, Sass J, Acaba J (2017). Ensuring inclusion of adolescent key populations at higher risk of HIV exposure: recommendations for conducting biological behavioral surveillance surveys. JMIR Public Heal Surveill.

[CR15] Kurth AE, Lally MA, Choko AT (2015). HIV testing and linkage to services for youth. J Int AIDS Soc.

[CR16] Mark D, Armstrong A, Andrade C (2017). HIV treatment and care services for adolescents: a situational analysis of 218 facilities in 23 sub-Saharan African countries. J Int AIDS Soc.

[CR17] Mmari K, Astone N (2014). Urban adolescent sexual and reproductive health in low-income and middle-income countries. Arch Dis Child.

[CR18] Musyoki H, Bhattacharjee P, Blanchard AK (2018). Changes in HIV prevention programme outcomes among key populations in Kenya: data from periodic surveys. PLoS ONE.

[CR19] Muthengi E, Gitau T, Austrian K (2016). Is working risky or protective for married adolescent girls in urban slums in Kenya? Understanding the association between working status, savings and intimate-partner violence. PLoS ONE.

[CR20] Mutumba M, Harper GW (2015). Mental health and support among young key populations: an ecological approach to understanding and intervention. J Int AIDS Soc.

[CR21] NACC (2015) Kenya aids strategic framework 2014/2015–2018/2019

[CR22] National AIDS and STI Control Programme (NASCOP) & Kenya Medical Research Institute (KEMRI) (2015) Guidelines for conducting adolescent HIV sexual and reproductive health research in Kenya. Gov Kenya 48

[CR23] Nkansah-Amankra S, Walker AD (2012). The relation between adolescent self assessment of health and risk behaviours: could a global measure of health provide indications of health risk exposures?. Health Educ J.

[CR24] Shabani O, Moleki MM, Thupayagale-Tshweneagae GGB (2018). Individual determinants associated with utilisation of sexual and reproductive health care services for HIV and AIDS prevention by male adolescents. Curationis.

[CR25] Ssewanyana D, Mwangala PN, Marsh V (2018). Young people’s and stakeholders’ perspectives of adolescent sexual risk behavior in Kilifi County, Kenya: a qualitative study. J Health Psychol.

[CR26] UNAIDS (2018) UNAIDS data 2018, pp 1–376

[CR27] Vitek CR, Čakalo JI, Kruglov YV (2014). Slowing of the HIV epidemic in Ukraine: evidence from case reporting and key population surveys, 2005–2012. PLoS ONE.

[CR28] Wanje G, Masese L, Avuvika E (2017). Parents’ and teachers’ views on sexual health education and screening for sexually transmitted infections among in-school adolescent girls in Kenya: a qualitative study. Reprod Health.

[CR29] World Health Organization (2014) Consolidated guidelines on HIV prevention, diagnosis, treatment and care for key populations. WHO Guideline 184 25996019

[CR30] Ziraba A, Orindi B, Muuo S (2018). Understanding HIV risks among adolescent girls and young women in informal settlements of Nairobi, Kenya: lessons for DREAMS. PLoS ONE.

